# Supplementary material for: A Critical View of the Application of the APEX Software (Aqueous Photochemistry of Environmentally-Occurring Xenobiotics) to Predict Photoreaction Kinetics in Surface Freshwaters
Source: Molecules. 2019 Dec 18;25(1):9. doi: 10.3390/molecules25010009 (PMC7017383; doi:10.3390/molecules25010009)
Supplement: Supplementary file 1 [file molecules-25-00009-s001.zip › 3.2.4_gcc-4.4.0.zip!/3.2.4_gcc-4.4.0/3.2.4_gcc-4.4.0/doc/octave/html/interpreter/Test-Functions.html]

Test Functions - Untitled


Next: Demonstration Functions,
Up: Test and Demo Functions


---

### B.1 Test Functions

— Function File: **test** name  
— Function File: **test** name quiet|normal|verbose  
— Function File: **test** ('name', 'quiet|normal|verbose', fid)  
— Function File: **test** ([], 'explain', fid)  
— Function File: success = **test** (...)  
— Function File: [n, max] = **test** (...)  
— Function File: [code, idx] = **test** ('name','grabdemo')  
> Perform tests from the first file in the loadpath matching name.
> `test` can be called as a command or as a function. Called with
> a single argument name, the tests are run interactively and stop
> after the first error is encountered.
>
> With a second argument the tests which are performed and the amount of
> output is selected.
>
> 'quiet': Don't report all the tests as they happen, just the errors. 'normal': Report all tests as they happen, but don't do tests which require user interaction. 'verbose': Do tests which require user interaction.
>
> The argument fid can be used to allow batch processing. Errors
> can be written to the already open file defined by fid, and
> hopefully when Octave crashes this file will tell you what was happening
> when it did. You can use `stdout` if you want to see the results as
> they happen. You can also give a file name rather than an fid, in
> which case the contents of the file will be replaced with the log from
> the current test.
>
> Called with a single output argument success, `test` returns
> true if all of the tests were successful. Called with two output arguments
> n and max, the number of successful tests and the total number
> of tests in the file name are returned.
>
> If the second argument is the string 'grabdemo', the contents of the demo
> blocks are extracted but not executed. Code for all code blocks is
> concatenated and returned as code with idx being a vector of
> positions of the ends of the demo blocks.
>
> If the second argument is 'explain', then name is ignored and an
> explanation of the line markers used is written to the file fid.
>
>
>
> **See also:** error, assert, fail, demo, example.

`test` scans the named script file looking for lines which
start with `%!`. The prefix is stripped off and the rest of the
line is processed through the Octave interpreter. If the code
generates an error, then the test is said to fail.

Since `eval()` will stop at the first error it encounters, you must
divide your tests up into blocks, with anything in a separate
block evaluated separately. Blocks are introduced by the keyword
`test` immediately following the `%!`. For example,

```
        %!test error ("this test fails!");
        %!test "test doesn't fail. it doesn't generate an error";
```

When a test fails, you will see something like:

```
          ***** test error ('this test fails!')
        !!!!! test failed
        this test fails!
```

Generally, to test if something works, you want to assert that it
produces a correct value. A real test might look something like

```
        %!test
        %! a = [1, 2, 3; 4, 5, 6]; B = [1; 2];
        %! expect = [ a ; 2*a ];
        %! get = kron (b, a);
        %! if (any(size(expect) != size(get)))
        %!    error ("wrong size: expected %d,%d but got %d,%d",
        %!           size(expect), size(get));
        %! elseif (any(any(expect!=get)))
        %!    error ("didn't get what was expected.");
        %! endif
```

To make the process easier, use the `assert` function. For example,
with `assert` the previous test is reduced to:

```
        %!test
        %! a = [1, 2, 3; 4, 5, 6]; b = [1; 2];
        %! assert (kron (b, a), [ a; 2*a ]);
```

`assert` can accept a tolerance so that you can compare results
absolutely or relatively. For example, the following all succeed:

```
        %!test assert (1+eps, 1, 2*eps)          # absolute error
        %!test assert (100+100*eps, 100, -2*eps) # relative error
```

You can also do the comparison yourself, but still have assert
generate the error:

```
        %!test assert (isempty([]))
        %!test assert ([ 1,2; 3,4 ] > 0)
```

Because `assert` is so frequently used alone in a test block, there
is a shorthand form:

```
        %!assert (...)
```

which is equivalent to:

```
        %!test assert (...)
```

Sometimes during development there is a test that should work but is
known to fail. You still want to leave the test in because when the
final code is ready the test should pass, but you may not be able to
fix it immediately. To avoid unnecessary bug reports for these known
failures, mark the block with `xtest` rather than `test`:

```
        %!xtest assert (1==0)
        %!xtest fail ('success=1','error'))
```

Another use of `xtest` is for statistical tests which should
pass most of the time but are known to fail occasionally.

Each block is evaluated in its own function environment, which means
that variables defined in one block are not automatically shared
with other blocks. If you do want to share variables, then you
must declare them as `shared` before you use them. For example, the
following declares the variable a, gives it an initial value (default
is empty), then uses it in several subsequent tests.

```
        %!shared a
        %! a = [1, 2, 3; 4, 5, 6];
        %!assert (kron ([1; 2], a), [ a; 2*a ]);
        %!assert (kron ([1, 2], a), [ a, 2*a ]);
        %!assert (kron ([1,2; 3,4], a), [ a,2*a; 3*a,4*a ]);
```

You can share several variables at the same time:

```
        %!shared a, b
```

You can also share test functions:

```
        %!function a = fn(b)
        %!  a = 2*b;
        %!assert (a(2),4);
```

Note that all previous variables and values are lost when a new
shared block is declared.

Error and warning blocks are like test blocks, but they only succeed
if the code generates an error. You can check the text of the error
is correct using an optional regular expression `<pattern>`.
For example:

```
        %!error <passes!> error('this test passes!');
```

If the code doesn't generate an error, the test fails. For example,

```
        %!error "this is an error because it succeeds.";
```

produces

```
        ***** error "this is an error because it succeeds.";
        !!!!! test failed: no error
```

It is important to automate the tests as much as possible, however
some tests require user interaction. These can be isolated into
demo blocks, which if you are in batch mode, are only run when
called with `demo` or `verbose`. The code is displayed before
it is executed. For example,

```
        %!demo
        %! t=[0:0.01:2*pi]; x=sin(t);
        %! plot(t,x);
        %! you should now see a sine wave in your figure window
```

produces

```
        > t=[0:0.01:2*pi]; x=sin(t);
        > plot(t,x);
        > you should now see a sine wave in your figure window
        Press <enter> to continue:
```

Note that demo blocks cannot use any shared variables. This is so
that they can be executed by themselves, ignoring all other tests.

If you want to temporarily disable a test block, put `#` in place
of the block type. This creates a comment block which is echoed
in the log file, but is not executed. For example:

```
        %!#demo
        %! t=[0:0.01:2*pi]; x=sin(t);
        %! plot(t,x);
        %! you should now see a sine wave in your figure window
```

Block type summary:

`%!test`: check that entire block is correct `%!error`: check for correct error message `%!warning`: check for correct warning message `%!demo`: demo only executes in interactive mode `%!#`: comment: ignore everything within the block `%!shared x,y,z`: declares variables for use in multiple tests `%!function`: defines a function value for a shared variable `%!assert (x, y, tol)`: shorthand for %!test assert (x, y, tol)

You can also create test scripts for builtins and your own C++
functions. Just put a file of the function name on your path without
any extension and it will be picked up by the test procedure. You
can even embed tests directly in your C++ code:

```
        #if 0
        %!test disp('this is a test')
        #endif
```

or

```
        /*
        %!test disp('this is a test')
        */
```

but then the code will have to be on the load path and the user
will have to remember to type test('name.cc'). Conversely, you
can separate the tests from normal Octave script files by putting
them in plain files with no extension rather than in script files.


— Function File: **assert** (cond)  
— Function File: **assert** (cond, errmsg, ...)  
— Function File: **assert** (cond, msg\_id, errmsg, ...)  
— Function File: **assert** (observed,expected)  
— Function File: **assert** (observed,expected,tol)  
> Produces an error if the condition is not met. `assert` can be
> called in three different ways.
>
> `assert (`cond`)` `assert (`cond`,` errmsg`, ...)` `assert (`cond`,` msg\_id`,` errmsg`, ...)`: Called with a single argument cond, `assert` produces an error if cond is zero. If called with a single argument a generic error message. With more than one argument, the additional arguments are passed to the `error` function. `assert (`observed`,` expected`)`: Produce an error if observed is not the same as expected. Note that observed and expected can be strings, scalars, vectors, matrices, lists or structures. `assert(`observed`,` expected`,` tol`)`: Accept a tolerance when comparing numbers. If tol is positive use it as an absolute tolerance, will produce an error if `abs(`observed `-` expected`) > abs(`tol`)`. If tol is negative use it as a relative tolerance, will produce an error if `abs(`observed `-` expected`) > abs(`tol `*` expected`)`. If expected is zero tol will always be used as an absolute tolerance.
>
>
>
>
> **See also:** test.

— Function File: **fail** (code,pattern)  
— Function File: **fail** (code,'warning',pattern)  
> Return true if code fails with an error message matching
> pattern, otherwise produce an error. Note that code
> is a string and if code runs successfully, the error produced is:
>
> ```
>                     expected error but got none
> ```
>
> If the code fails with a different error, the message produced is:
>
> ```
>                     expected <pattern>
>                     but got <text of actual error>
> ```
>
> The angle brackets are not part of the output.
>
> Called with three arguments, the behavior is similar to
> `fail(`code`,` pattern`)`, but produces an error if no
> warning is given during code execution or if the code fails.
